# The Protective Effect of Indole-3-Acetic Acid (IAA) on H_2_O_2_-Damaged Human Dental Pulp Stem Cells Is Mediated by the AKT Pathway and Involves Increased Expression of the Transcription Factor Nuclear Factor-Erythroid 2-Related Factor 2 (Nrf2) and Its Downstream Target Heme Oxygenase 1 (HO-1)

**DOI:** 10.1155/2017/8639485

**Published:** 2017-06-14

**Authors:** Daehwan Kim, Hyewon Kim, Kichul Kim, Sangho Roh

**Affiliations:** Cellular Reprogramming and Embryo Biotechnology Laboratory, Dental Research Institute, BK21, Seoul National University School of Dentistry, Seoul, Republic of Korea

## Abstract

Indole-3-acetic acid (IAA) is the most common plant hormone of the auxin class and is known to have many effects including cell proliferation enhancement and antioxidant property. However, no study has revealed its defensive effects against oxidative toxicity in human dental pulp stem cells (hDPSCs). In this study, we investigated the effects of IAA on hydrogen peroxide- (H_2_O_2_-) induced oxidative toxicity in hDPSCs. H_2_O_2_-induced cytotoxicity was attenuated after IAA treatment. Cell cycle analysis using FACS showed that the damaged cell cycle and increased number of apoptotic cells by H_2_O_2_ treatment were recovered after the treatment of IAA. The H_2_O_2_-mediated increased expression of the proapoptotic genes, *BAX* and *p53*, was attenuated by IAA treatment, while IAA treatment increased antiapoptotic genes, *BCL-2* and *ATF5* expression. The increases of cleaved caspase-3 and ROS by H_2_O_2_ were also decreased after treatment of IAA. To further investigate the mechanism of IAA, Nrf2-related antioxidant pathway was examined and the results showed that the level of Nrf2 and HO-1 expressions, stimulated by H_2_O_2,_ decreased after treatment of IAA. Moreover, IAA treatment protected hDPSCs against H_2_O_2_-induced oxidative stress via increased expression of Nrf2 and HO-1, mediated by the AKT pathway.

## 1. Introduction

Dental pulp stem cells (DPSCs) are adult stem cells (ASCs) that are able to differentiate into multiple lineages [1]. Although there is one report published on teratoma-like structures from DPSCs [2], the cells in general condition are still regarded as fascinating ASCs without tumorigenesis [3]. Normally, DPSCs can be isolated from various teeth including permanent teeth and supernumerary teeth [4, 5]. The characteristics of DPSCs are similar to those of bone marrow-derived MSCs (BMSCs) [6, 7]. It has been reported that DPSCs have the potential to differentiate into mesenchymal lineages including odontoblasts, chondrocytes, myocytes, adipocytes, and osteoblasts [6, 7] as well as nonmesenchymal ectodermal lineages, which include neurons [8]. DPSCs are therefore regarded as an alternative source of BMSCs. Moreover, the isolation and cultivation of DPSCs are easier than those of BMSCs, and their proliferation rate is higher [5, 9]. The fact that nonfunctional or useless supernumerary teeth can be sources for DPSCs makes them a noninvasive alternative to BMSCs.

Auxins are plant hormones that have many different functions including growth, development, and wound response [10, 11]. Recently, it has been demonstrated that auxins are able to regulate senescence in plants [12, 13]. Moreover, some auxins also have antioxidant activities in plants [14, 15]. Indole-3-acetic acid (IAA) is one of the most important members of the auxins and is synthesized naturally by plants [16]. It has been confirmed that IAA is present not only in plants but also in animals, including mammals [17, 18]. However, only a few studies have examined the functions of IAA in humans. Moreover, to date, no studies have delineated the effects of IAA on hDPSCs.

Hydrogen peroxide (H_2_O_2_) is a powerful inducer of oxidative stress, which causes endothelial cell dysfunction, cellular injury, and vascular disease [19–21]. H_2_O_2_ can also cause cell senescence and induce apoptosis [22, 23]. In the dental field, H_2_O_2_ is generally used for tooth whitening both professionally and in self-administered products (up to 35%) in its original form or in the form of carbamide peroxide [24, 25]. As a result of the demand for products that improve appearance, H_2_O_2_ tooth bleaching has become popular. However, adverse effects such as cervical root resorption, tooth sensitivity, ulceration of soft tissue, and potential tumor promotion can occur [26–28]. Moreover, it has been demonstrated that H_2_O_2_ can penetrate enamel and dentin, resulting in damage to dental pulp cells [29, 30]. However, little is known about the effect of H_2_O_2_ on hDPSCs. Moreover, the effects of IAA on H_2_O_2_-induced damage and the mechanism of its action in hDPSCs have not been elucidated.

In the present study, we investigated the effects of IAA on hDPSCs during H_2_O_2_-induced oxidative toxicity. More specifically, we determined if this compound protected hDPSCs from apoptotic and oxidative stress by assessing hDPSC morphology, proliferation, survival, cell cycle, and gene expression patterns.

## 2. Materials and Methods

### 2.1. Chemicals

Most inorganic and organic compounds were purchased from Sigma-Aldrich Korea (Yong-in, Korea), and all liquid medium and supplements were from Life Technologies (Grand Island, NY, USA) unless indicated otherwise in the text.

### 2.2. Human Dental Pulp Cell Culture

According to guidelines provided by the Institutional Review Board (IRB, number S-D20100005), human maxillary central supernumerary teeth (*N* = 8) were extracted from children at the Dental Hospital of Seoul National University. Human DPSC culture process from isolation of pulp tissue to passaging culture followed our laboratory protocol [31]. Briefly, the cementoenamel junction was cut by a cutting disk to expose the pulp tissue as described previously [32] and pulp tissue was gently separated using a sterile endodontic file. After enzymatic dissociation with 1% (*w/v*) collagenase type I, single-cell suspensions were seeded into 24-well culture dishes. Then, the cells were incubated at 37°C in a humidified atmosphere containing 5% CO_2_ in DPSC culture medium, which consisted of *α*-MEM supplemented with 10% (*v/v*) fetal bovine serum (FBS; Life Technologies). Culture medium was replaced every three days, and they were subcultured at one-fifth dilution for later passaging when the cells were grown to 70% confluence. To avoid the use of senescent cells, all experiments were performed on cultured cells of passage number 3 to 5.

### 2.3. Cell Viability Assay

After H_2_O_2_ and/or IAA treatment, the number of viable cells was determined by a 3-(4,5-dimethylthiazol-2-yl)-5-(3-carboxymethoxyphenyl)-2-(4-sulfophenyl)-2H-tetrazolium (MTS) assay using the commercially available CellTiter 96® Aqueous Non-Radioactive Cell Proliferation Assay (Promega, Madison, WI). hDPSCs were cultured in a 96-well plate at a cell density of 1 × 10^4^ and treated with H_2_O_2_ for 24 hours before performing the MTS assay.

### 2.4. Cell Cycle Analysis

After H_2_O_2_ and/or IAA treatment, cells were detached with trypsin and collected. They were resuspended and fixed in 70% ethanol at −20°C for 30 minutes. After cells were centrifuged and washed with PBS, 350 *μ*l of propidium iodide (PI, 40 *μ*g/ml) was added for cell staining and then 2 *μ*l of RNase A was added. After staining, the PI-elicited fluorescence of individual cells was determined by flow cytometry (FACSAria1®; BD Biosciences, Erembodegem, Belgium). The total amount of PI fluorescence of 1 × 10^4^ cells was counted in each sample. The distribution of cells in G0/G1, S, and G2/M phase was calculated using the ModFit LT program version 3.3 (Verity Software House Inc., USA).

### 2.5. Real-Time Polymerase Chain Reaction (PCR)

Total RNA from samples was extracted using the RNeasy mini kit (Qiagen, Hilden, Germany), and M-MLV Reverse Transcriptase was used to synthesize cDNA according to the manufacturer's instructions. Real-time PCR was performed using the 7500HT system™ (Applied Biosystems Inc., Foster City, CA, USA) and SYBR Premix Ex Taq II (Takara, Otsu, Japan). The PCR volume was 20 *μ*l, and 1 *μ*l reverse transcript product was used. Cycling conditions were as follows: 1 cycle of 95°C for 30 s, 40 cycles of 95°C for 5 s, and 60°C for 30 s. The ΔΔCt method was used for relative quantitation of mRNA expression in samples, and the fold change was determined as 2^−ΔΔCt^. Specific primer sequences to amplify apoptotic marker genes are from previous reports and are listed in Table 1 [33, 34].

### 2.6. Measurement of Reactive Oxygen Species (ROS)

A DCF-DA cellular ROS detection assay (Abcam PLC, Cambridge, MA, USA) was used to measure hydroxyl, peroxyl, and other ROS activity within cells. A total of 2.5 × 10^4^ cells per well were seeded on a 96-well plate and allowed to attach for 24 h. Cells were then stained with 25 *μ*M DCF-DA for 45 min at 37°C. After staining, cells were treated with H_2_O_2_ and/or IAA for 6 h. Finally, fluorescent intensity was determined by fluorescence spectroscopy with maximum excitation and emission spectra of 485 and 535 nm, respectively.

### 2.7. Western Blot Analysis

Cells were lysed in passive lysis buffer (Promega) and harvested with a cell scraper. Cell debris was removed by centrifuging the cell lysate at 13,000 rpm for 10 minutes at 4°C, and 30 *μ*g of proteins were loaded on 10% SDS-PAGE gels and separated by gel electrophoresis. Proteins were then transferred to polyvinylidene fluoride (PVDF) membranes (Millipore, Billerica, MA, USA) and blocked for 1 h with 10% nonfat milk in Tris-buffered saline with 0.1% Tween 20. Proteins were then blotted with antibodies against Nrf2 (C-20, Santa Cruz Biotechnology, Santa Cruz, CA), HO-1 (H-105, Santa Cruz Biotechnology), lamin B1 (A-11, Santa Cruz Biotechnology), cleaved caspase-3 (9661, Cell Signaling, Beverly, MA), and beta-actin (C4, Santa Cruz Biotechnology). Detection of the primary antibody was accomplished using HRP-conjugated anti-mouse IgG (1 : 3000, Santa Cruz Biotechnology) and anti-rabbit IgG (1 : 3000, Santa Cruz Biotechnology). Intensities of the protein bands were evaluated by densitometric analysis using GeneGnome XRQ (Syngene Corp., Cambridge, UK).

### 2.8. Statistical Analysis

All values are expressed as means ± SDs. To determine the significance of differences among groups, comparisons were made using Student's *t*-test as implemented in GraphPad Prism V5.0 (GraphPad Software, San Diego, CA, USA). *P* < 0.05 was considered significant.

## 3. Results

### 3.1. The Effect of H_2_O_2_ and IAA on the Viability of hDPSCs

**Figure 1:**
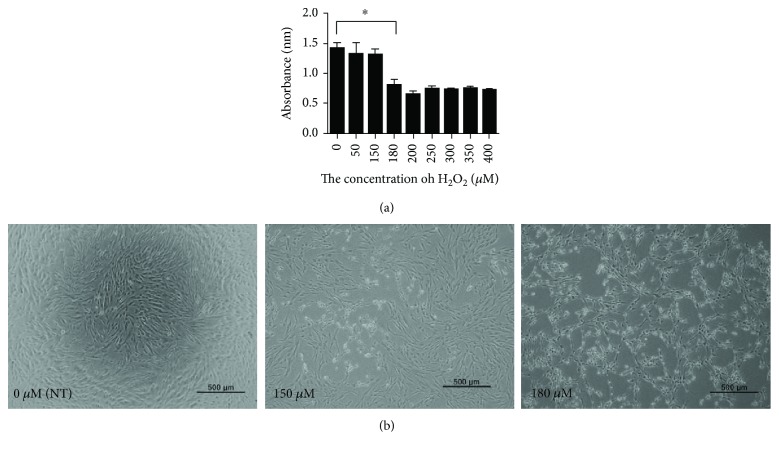
Effect of H_2_O_2_ on human dental pulp stem cells (hDPSCs). (a) The viability of hDPSCs was also assessed after treating the cells with different concentrations of H_2_O_2_ ranging from 1 to 400 *μ*M. The viability of hDPSCs decreased after treatment with 180 *μ*M and higher concentrations of H_2_O_2_. (b) Morphologies of hDPSCs cultured with 0 *μ*M (nontreated group, NT), 150 *μ*M, and 180 *μ*M H_2_O_2_. *N* = 4, ^∗^*P* < 0.0001.

To examine the effects of H_2_O_2_ on hDPSCs, cells were exposed to different concentrations of H_2_O_2_ in the culture medium for 24 h. As shown in Figure 1, H_2_O_2_ concentrations of less than 180 *μ*M had no effect on cell viability (Figure 1(a)). However, the viability of hDPSCs significantly decreased after treatment with H_2_O_2_ at 180 *μ*M and above compared with the nontreated (NT) group. The morphology of hDPSCs was also analyzed, and the results were in agreement with the cell viability findings. The shape of hDPSCs was not significantly changed by H_2_O_2_ at 1–150 *μ*M concentrations (Figure 1(b)). However, many hDPSCs condensed and detached (Figure 1(b)) when cells were treated with 180 *μ*M H_2_O_2_. Cells in the model group were therefore subsequently treated with 180 *μ*M H_2_O_2_.

**Figure 2:**
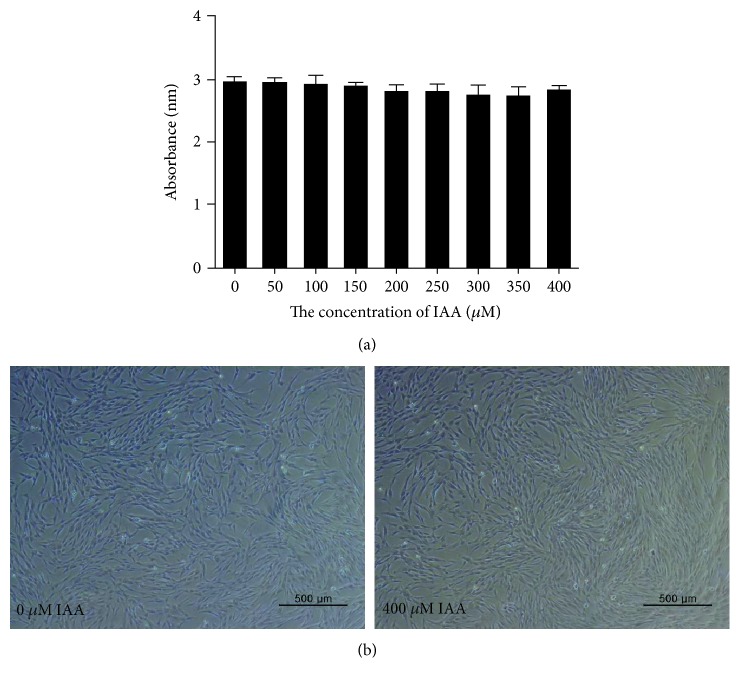
The effect of IAA on human dental pulp stem cells (hDPSCs). (a) The viability of hDPSCs was measured after treatment with IAA at different concentrations ranging from 1 to 400 *μ*M. (b) The morphologies of hDPSCs cultured with 0 *μ*M and 400 *μ*M IAA. *N* = 4.

The potential cytotoxic effects of IAA were measured after treatment of hDPSCs with different concentrations of IAA ranging from 1 to 400 *μ*M. Cell viability of hDPSCs was not influenced by IAA treatment, and cells did not condense or detach (Figures 2(a) and 2(b)).

### 3.2. Protective Effect of IAA against H_2_O_2_-Induced hDPSC Damage

**Figure 3:**
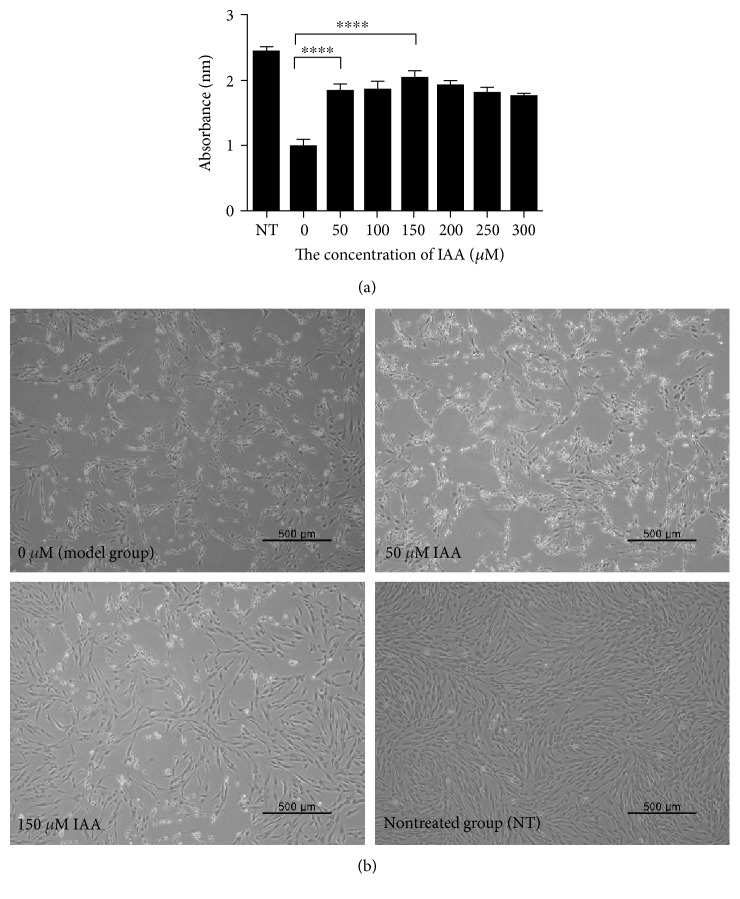
The protective effect of IAA against H_2_O_2_-induced oxidative damage of hDPSCs. (a) Viability analysis of hDPSCs damaged by 180 *μ*M H_2_O_2_ after IAA treatment. IAA treatment significantly increased cell and was maximal at 150 *μ*M IAA. (b) H_2_O_2_-damaged hDPSCs cultured with 0 *μ*M, 50 *μ*M, and 150 *μ*M IAA. *N* = 4, ^∗∗∗∗^*P* < 0.0001.

To evaluate whether IAA protected against H_2_O_2_-induced cytotoxicity, the cell viability and morphology of H_2_O_2_-damaged hDPSCs were assessed after treatment with IAA at various concentrations ranging from 1 to 300 *μ*M. H_2_O_2_-induced cytotoxicity was significantly attenuated in the presence of IAA, with the maximum effect observed at 150 *μ*M IAA (Figure 3(a)). In addition, the number of condensed and floating H_2_O_2_-damaged hDPSCs significantly decreased after treatment with 150 *μ*M IAA compared with the model group (Figure 3(b)).

### 3.3. IAA Treatment Rescues the Cell Cycle and Prevents Apoptosis of H_2_O_2_-Damaged hDPSCs

**Figure 4:**
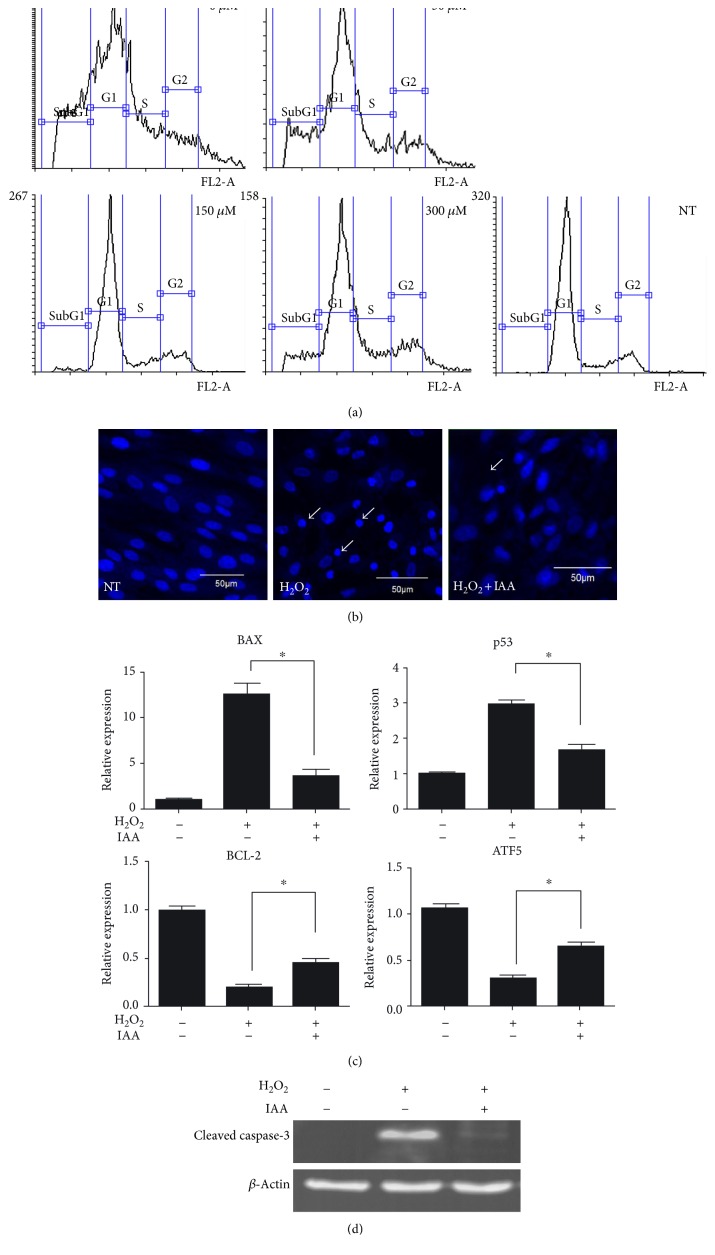
IAA treatment suppresses H_2_O_2_-induced cytotoxicity via regulation of the cell cycle and apoptosis in human dental pulp stem cells (hDPSCs). (a) Analysis of cell cycle after IAA treatment of H_2_O_2_-damaged hDPSCs. (b) DNA staining with DAPI. The number of cells with DNA condensation (arrow) decreased after IAA treatment of H_2_O_2_-damaged hDPSCs. Expression of the apoptosis-related genes *BAX*, *p53*, *BCL-2*, and *ATF5* was evaluated by real-time PCR (c) and the expression of the apoptosis-related enzyme, cleaved caspase-3, was evaluated by Western blot (d). *N* = 3, ^∗^*P* < 0.001.

To further analyze the protective effect of IAA on H_2_O_2_-induced cytotoxicity, cell cycle was quantified by flow cytometry after PI staining of cells (Figure 4(a)). In the model group, the normal cell cycle was notably disrupted by H_2_O_2_ treatment compared with the NT group. In particular, the number of apoptotic cells (sub-G1) was significantly increased compared to the NT group. Cell cycle disruption by H_2_O_2_-induced cytotoxicity was rescued by treatment with 150 *μ*M IAA, and the number of apoptotic cells was also decreased by IAA treatment.

To determine the effects of IAA on H_2_O_2_-induced apoptosis, additional analyses were conducted. Firstly, DAPI-stained nuclei were observed. In the model group, nuclear condensation and chromatin margination, which are typical properties of apoptotic cells (Figure 4(b)), were evident. However, the number of condensed nuclei was significantly lower in the IAA-treated group than in the model group.

The expression of apoptotic and antiapoptotic genes was also assessed by real-time PCR. Expression of the proapoptotic genes *BAX* and *p53* was significantly increased by H_2_O_2_ treatment. However, the expression of *BCL-2* and *ATF5*, which are antiapoptotic genes, was significantly lower in the model group than in the NT group (Figure 4(c)). The H_2_O_2_-mediated increase in *BAX* and *p53* expression was significantly attenuated by IAA treatment, while IAA treatment increased *BCL-2* and *ATF5* expression. We also assessed the expression of cleaved caspase-3 by Western blot (Figure 4(d)). Levels of cleaved caspase-3 were higher in the model group than in the NT group, while the expression of cleaved caspase-3 was noticeably decreased in the IAA-treated group than in the model group.

### 3.4. IAA Treatment Suppresses the Generation of Reactive Oxygen Species (ROS)

**Figure 5:**
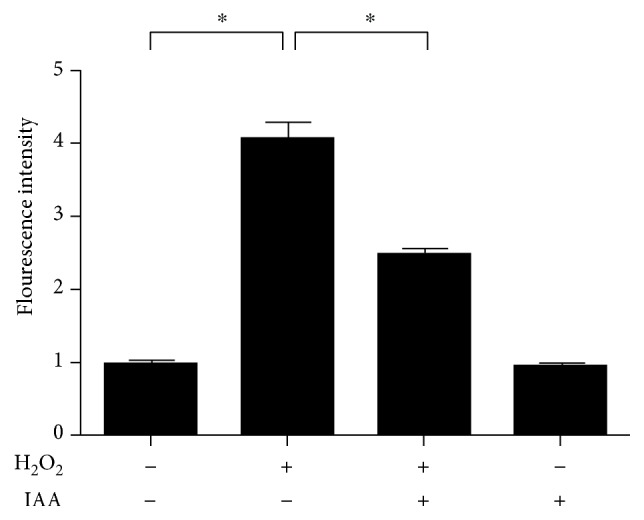
Analysis of ROS levels in human dental pulp stem cells (hDPSCs) after sequential treatment with H_2_O_2_ and IAA. ROS level was significantly higher in the model group than in the nontreated group. However, the ROS level in the model group was noticeably decreased after treatment with IAA. IAA treatment alone had no effect on ROS levels in hDPSCs. *N* = 4, ^∗^*P* < 0.001.

To investigate the effects of IAA on the generation of ROS, we examined intracellular ROS levels in hDPSCs using DCF-DA. The fluorescence intensity of DCF-DA was significantly higher in the model group than in the NT group (Figure 5). In contrast, the fluorescence intensity of DCF-DA was dramatically lower in the IAA-treated group than in the model group.

### 3.5. IAA Treatment Induces the Expression of Nuclear Factor-Erythroid 2-Related Factor 2 (Nrf2) via AKT Signaling

**Figure 6:**
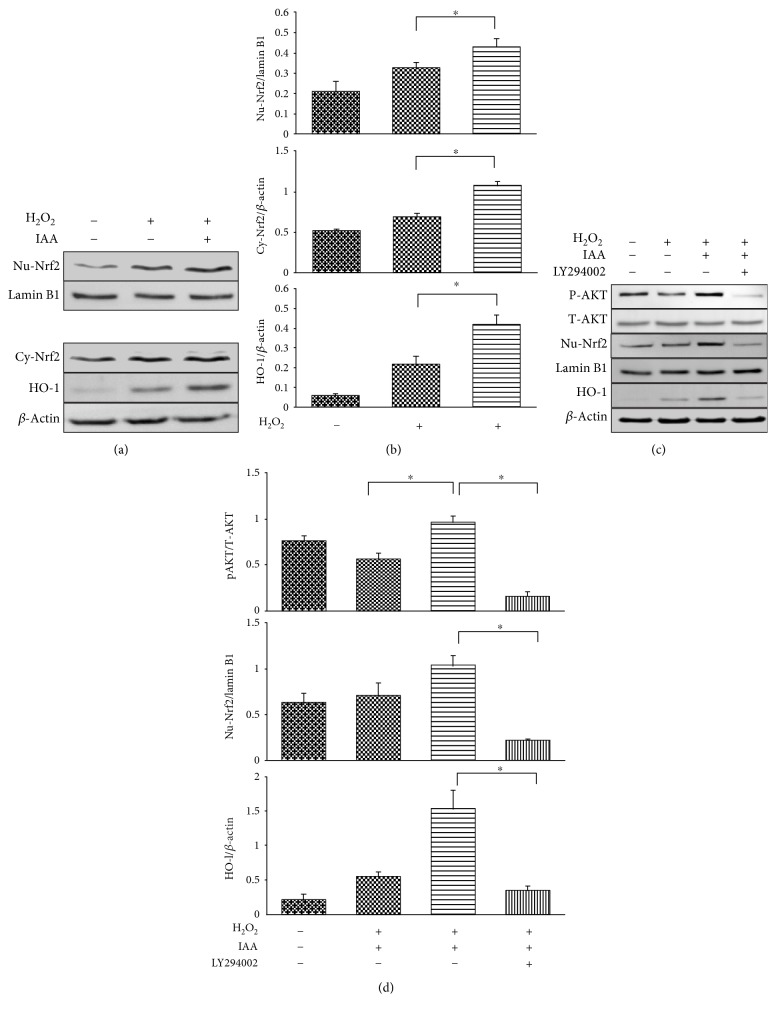
Effects of IAA on nuclear factor erythroid 2-related factor 2 (Nrf2) and heme oxygenase 1 (HO-1) and the role of AKT signaling in human dental pulp stem cells (hDPSCs). (a) The expression of Nrf2 was analyzed in cytosolic (Cy) and nuclear (Nu) extracts from cells. The antioxidant enzyme, HO-1, was also profiled after treatment of H_2_O_2_-damaged hDPSCs with IAA. (b) Densitometric analysis of Cy-Nrf2, Nu-Nrf2, and HO-1 expression. (c) Roles of AKT signaling in IAA-induced Nu-Nrf2 and HO-1 expression. The IAA-induced increase in expression of pAKT, Nu-Nrf2, and HO-1 was significantly reduced by treatment with the AKT inhibitor LY294002. (d) Densitometric analysis of pAKT, Nu-Nrf2, and HO-1 expression. ^∗^*P* < 0.05, *N* = 4.

Nrf2 is a key transcription factor involved in the regulation of antioxidant genes; we therefore assessed expression of Nrf2 in cytosolic and nuclear fractions by Western blot. Cytosolic Nrf2 expression was significantly higher in the IAA-treated group than in the model group (Figures 6(a) and 6(b)). The pattern of nuclear Nrf2 expression was analogous to that observed for cytosolic Nrf2. Interestingly, heme oxygenase 1 (HO-1), an antioxidant enzyme regulated by Nrf2, was expressed at higher levels in the IAA-treated group than in the model group (Figures 6(a) and 6(b)).

To further investigate the mechanisms underlying increased Nrf2 expression in the IAA-treated group, levels of phosphorylated AKT (pAKT) after IAA treatment were evaluated. pAKT expression was remarkably higher in the IAA-treated group than in the model group (Figures 6(c) and 6(d)). Treatment of cells with LY294002, an AKT inhibitor, significantly decreased the expression of pAKT in the IAA-treated group (Figures 6(c) and 6(d)). Interestingly, Nrf2 and HO-1 expression was also significantly decreased in the IAA-treated group after treatment with LY294002, consistent with the pAKT expression pattern described above (Figures 6(c) and 6(d)).

## 4. Discussion

Because of the increased demand for aesthetic treatments in the dental field, H_2_O_2_-mediated dental bleaching procedures are commonly performed. During dental treatment, the H_2_O_2_ is able to permeate into dental pulp tissue through dentin [30, 35] and H_2_O_2_-induced oxidative stress will damage the tissue. Furthermore, it has been demonstrated that oxidative stress is significantly related to chronic apical periodontitis [36]. However, the effect of H_2_O_2_-induced oxidative stress on hDPSCs has received little attention.

In the present study, we confirmed that the treatment of IAA, a plant hormone, protected hDPSCs from H_2_O_2_-induced damage, including oxidative stress and apoptosis. In addition, the effects of IAA on the oxidative stress pathway in hDPSCs were demonstrated.

To evaluate the effects of H_2_O_2_ on hDPSCs, we examined cell viability and morphology. The viability of hDPSCs treated with 180 *μ*M H_2_O_2_ was considerably lower than that of the NT group (Figure 1(b)). Many more condensed and detached cells were observed among hDPSCs treated with 180 *μ*M H_2_O_2_ than the NT group, suggesting that 180 *μ*M H_2_O_2_ is sufficient to establish an H_2_O_2_-damaged hDPSC model (Figure 1(b)). These findings are in agreement with a previous study [37]. In contrast to H_2_O_2_, IAA had no effect on the viability of hDPSCs, even at high concentrations (Figure 2). These results suggest that IAA is not toxic to hDPSCs at the concentrations evaluated.

To clarify the effect of IAA on H_2_O_2_-damaged hDPSCs, H_2_O_2_-damaged hDPSCs were treated with various concentrations of IAA. The viability of 150 *μ*M IAA-treated hDPSCs was significantly higher than that of the model group (180 *μ*M H_2_O_2_) (Figure 3(a)). Moreover, the number of condensed and detached cells was lower in the IAA-treated group (150 *μ*M IAA) than in the model group (Figure 3(b)). Moreover, living cells in the 150 *μ*M IAA-treated group had similar morphologies to those in the NT group. Together, these results indicate that IAA treatment can protect hDPSCs from H_2_O_2_-induced damage.

Previously, it was reported that the exposure of cells to H_2_O_2_ was able to trigger cell cycle arrest and apoptosis [38, 39]. We therefore hypothesized that IAA may prevent the H_2_O_2_-induced disruption of cell cycle and apoptosis in hDPSCs. In the model group, the cell cycle was completely disrupted compared to that in the NT group (Figure 4(a)). However, after treatment of cells with 150 *μ*M IAA, the cell cycle was rescued, suggesting that H_2_O_2_-induced damage of the cell cycle was restored by IAA. Interestingly, the population of sub-G1 cells was significantly higher in the NT group than in the model group. Cells in the sub-G1 range are considered to be dead cells, including necrotic and apoptotic cells [40]. Because H_2_O_2_ not only disrupts the cell cycle but also induces apoptosis [38, 39], we hypothesized that H_2_O_2_-induced apoptosis could be involved in disruption of the cell cycle, implying that the protective effect of IAA was also associated with apoptosis. As expected, hDPSCs with condensed and fragmented nuclei, suggesting apoptotic cells, were observed in the model group (Figure 4(b)). However, there were far fewer cells with condensed and fragmented nuclei in the IAA-treated group than in the model group. Expression of apoptosis-related genes was also evaluated; expression of proapoptotic genes was decreased whereas that of antiapoptotic genes was increased in the IAA-treated group compared to the model group (Figure 4(c)). Moreover, cleaved caspase-3 expression in the model group was restored after IAA treatment (Figure 4(d)). Treatment of hDPSCs with H_2_O_2_ therefore disrupted the cell cycle and induced apoptosis. However, IAA protected against H_2_O_2_-induced damage by rescuing the cell cycle and preventing apoptosis. To the best of our knowledge, this is the first study to demonstrate that IAA is able to protect hDPSCs from H_2_O_2_-induced damage through cell cycle- and apoptosis-related pathways.

Oxidative stress leads to cellular damage and death via generation of ROS [41]. We therefore hypothesized that IAA treatment would regulate the ROS pathway. To test this hypothesis, we evaluated the effects of IAA treatment on ROS generation. ROS levels were significantly higher in the model group than in the NT group (Figure 5). In contrast, ROS generation was significantly lower in the IAA-treated group than in the model group. These results suggest that IAA treatment influences ROS and oxidative stress pathways. However, IAA alone did not have any significant effects on the generation of ROS.

Nrf2 is a key transcription factor that regulates expression of endogenous antioxidant enzymes. Under normal conditions, Nrf2 is bound to Kelch-like epichlorohydrin-associated protein 1 (Keap-1), and this complex is located in the cytoplasm [42]. Under oxidative stress conditions, Nrf2 is released from Keap-1 and translocates to the nucleus, where it recognizes the antioxidant response element (ARE) and regulates the expression of antioxidant enzymes, including HO-1. Thus, the Nrf2-ARE pathway is crucial for protecting against oxidative stress. In previous studies, the Nrf2-ARE pathway has been shown to be highly activated to protect against oxidative damage [43]. In the present study, we also investigated whether IAA is able to regulate the Nrf2-ARE pathway. Treatment of hDPSCs with IAA resulted in enhanced expression of both Nrf2 and HO-1, suggesting that IAA treatment activates the Nrf2-ARE pathway, resulting in protection against H_2_O_2_-induced oxidative damage and enhanced cell viability (Figures 6(a) and 6(b)).

AKT signaling is involved in activation of the Nrf2-ARE pathway in response to oxidative stress [44]. AKT signaling also regulates cell survival via antioxidant and antiapoptotic roles. We found that pAKT expression was remarkably higher in the IAA-treated group than in the model group, while pAKT expression was noticeably decreased by treatment with LY294002 (Figures 6(c) and 6(d)). In addition, the pattern of Nrf2 and HO-1 expression was analogous to that of pAKT expression, implying that Nrf2 and HO-1 induced by IAA may be regulated by AKT signaling in H_2_O_2_-damaged hDPSCs. Together, these results suggest that IAA activates Nrf2 and HO-1 expression through AKT signaling, thereby protecting against oxidative damage.

**Figure 7:**
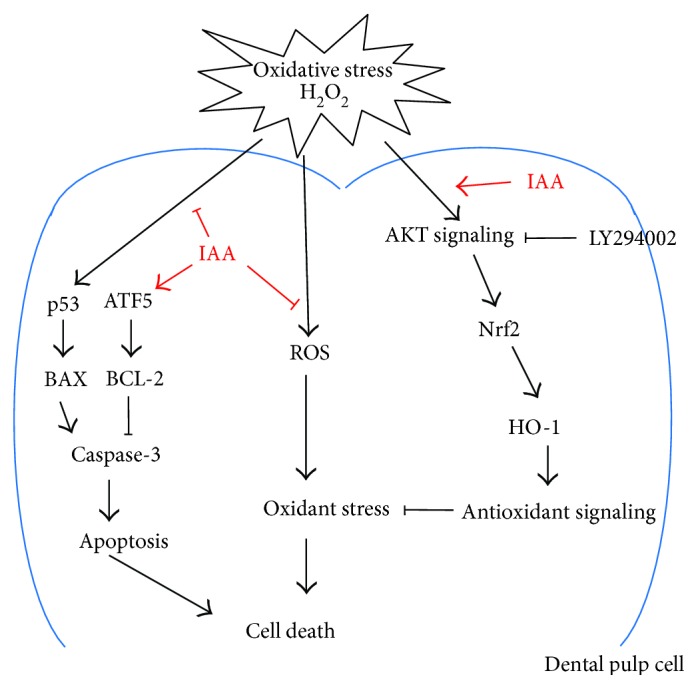
Proposed mechanisms of action of IAA in H_2_O_2_-exposed human dental pulp stem cells. IAA treatment may have an antiapoptotic effect and protect against H_2_O_2_-induced oxidative stress by increasing the expression of Nrf2 and HO-1, mediated by the AKT pathway.

In conclusion, we demonstrated that IAA treatment protected hDPSCs against H_2_O_2_-induced oxidative stress via increased expression of Nrf2 and HO-1, mediated by the AKT pathway (Figure 7). To the best of our knowledge, this is the first report of the protective effect of the natural plant hormone IAA, suggesting that application of this plant hormone may have therapeutic value in the treatment of human dental diseases associated with oxidative stress.

## Figures and Tables

**Table 1:** Primer sequences used in the real-time polymerase chain reaction experiments.

	Forward primer (5′-3′)	Reverse primer (5′-3′)
*BCL-2*	TTGTGGCCTTCTTTGAGTTCGGTG	GGTGCCGGTTCAGGTACTCAGTCA
*ATF5*	TATGAGGTCCTTGGGGGTG	ACCCGCTCAGTCATCCAAT
*BAX*	CCTGTGCACCAAGGTGCCGGAACT	CCACCCTGGTCTTGGATCCAGCCC
*p53*	CTAGCATTCAGGCCCTCATC	TCCGACTGTGACTCCTCCAT
*GAPDH*	GCTCTCTGCTCCTCCCTGTTCTAG	TGGTAACCAGGCGTCCGAT
